# Interrogation of living myocardium in multiple static deformation states with diffusion tensor and diffusion spectrum imaging

**DOI:** 10.1016/j.pbiomolbio.2014.08.002

**Published:** 2014-08

**Authors:** Maelene Lohezic, Irvin Teh, Christian Bollensdorff, Rémi Peyronnet, Patrick W. Hales, Vicente Grau, Peter Kohl, Jürgen E. Schneider

**Affiliations:** a British Heart Foundation Experimental Magnetic Resonance Unit, Division of Cardiovascular Medicine, Radcliffe Department of Medicine, University of Oxford, Oxford, UK; b National Heart and Lung Institute, Imperial College London, London, UK; c Qatar Cardiovascular Research Center, Qatar Foundation, Doha, Qatar; d Imaging and Biophysics Unit, Institute of Child Health, University College London, London, UK; e Department of Engineering Science, University of Oxford, Oxford, UK; f Department of Computer Science, University of Oxford, Oxford, UK

**Keywords:** Magnetic resonance imaging, Cardiac MRI, Langendorff perfusion, Isolated heart, Diffusion tensor imaging, Diffusion spectrum imaging

## Abstract

Diffusion tensor magnetic resonance imaging (MRI) reveals valuable insights into tissue histo-anatomy and microstructure, and has steadily gained traction in the cardiac community. Its wider use in small animal cardiac imaging *in vivo* has been constrained by its extreme sensitivity to motion, exaggerated by the high heart rates usually seen in rodents. Imaging of the isolated heart eliminates respiratory motion and, if conducted on arrested hearts, cardiac pulsation. This serves as an important intermediate step for basic and translational studies. However, investigating the micro-structural basis of cardiac deformation in the same heart requires observations in different deformation states.

Here, we illustrate the imaging of isolated rat hearts in three mechanical states mimicking diastole (cardioplegic arrest), left-ventricular (LV) volume overload (cardioplegic arrest plus LV balloon inflation), and peak systole (lithium-induced contracture). An optimised MRI-compatible Langendorff perfusion setup with the radio-frequency (RF) coil integrated into the wet chamber was developed for use in a 9.4T horizontal bore scanner. Signal-to-noise ratio improved significantly, by 75% compared to a previous design with external RF coil, and stability tests showed no significant changes in mean *T*_1_, *T*_2_ or LV wall thickness over a 170 min period. In contracture, we observed a significant reduction in mean fractional anisotropy from 0.32 ± 0.02 to 0.28 ± 0.02, as well as a significant rightward shift in helix angles with a decrease in the proportion of left-handed fibres, as referring to the locally prevailing cell orientation in the heart, from 24.9% to 23.3%, and an increase in the proportion of right-handed fibres from 25.5% to 28.4%. LV overload, in contrast, gave rise to a decrease in the proportion of left-handed fibres from 24.9% to 21.4% and an increase in the proportion of right-handed fibres from 25.5% to 26.0%. The modified perfusion and coil setup offers better performance and control over cardiac contraction states.

We subsequently performed high-resolution diffusion spectrum imaging (DSI) and 3D whole heart fibre tracking in fixed *ex vivo* rat hearts in slack state and contracture. As a model-free method, DSI augmented the measurements of water diffusion by also informing on multiple intra-voxel diffusion orientations and non-Gaussian diffusion. This enabled us to identify the transition from right- to left-handed fibres from the subendocardium to the subepicardium, as well as voxels in apical regions that were traversed by multiple fibres. We observed that both the mean generalised fractional anisotropy and mean kurtosis were lower in hearts in contracture compared to the slack state, by 23% and 9.3%, respectively. While its heavy acquisition burden currently limits the application of DSI *in vivo*, ongoing work in acceleration techniques may enable its use in live animals and patients. This would provide access to the as yet unexplored dimension of non-Gaussian diffusion that could serve as a highly sensitive marker of cardiac micro-structural integrity.

## 1 Introduction

Myocardial tissue structure is a key determinant of the mechanical and electrical properties of the heart in health and disease. Detailed characterisation of three-dimensional (3D) cardiac histo-architecture, and its dynamic alteration during the contractile cycle, offers great potential for new insights into the mechanics of cardiac contraction. Most studies of myocardial microstructure have been conducted on fixed samples, using tissue-destructive techniques such as serial histology sectioning. In contrast, diffusion magnetic resonance imaging (MRI) is a non-invasive tool that enables determination of 3D morphological features in the intact organ (Plank et al., 2009, Scollan et al., 1998).

Diffusion MRI is based on the random diffusion or Brownian motion of water molecules in response to thermal energy, the mechanism of which was first described by Einstein (Einstein, 1905). MRI data acquisition is typically sensitized to diffusion by adding pairs of magnetic field gradients to an imaging sequence (Stejskal and Tanner, 1965). The first gradient labels protons with a given phase, while the second gradient reverses it. Any diffusion that occurs along the axis of the gradients during the time interval between the pair of gradients leads to measurable signal attenuation due to imperfect reversal of phase, which is dependent on the water diffusivity, *D*, and the degree of diffusion weighting, described by the b-value (Le Bihan et al., 1991). The experiment is typically repeated with diffusion gradients of different strengths and orientations. As water diffusion is influenced by the presence of micro-anatomical structures, its measurement provides information on cell and tissue micro-architecture at a scale beyond the imaging resolution.

Several diffusion MRI methods have been employed in cardiac imaging. These vary considerably in terms of the minimum number of images required, acquisition time, post-processing and information obtained. For instance, trace imaging is a rapid technique that enables calculation of the mean apparent diffusion coefficient (ADC), which may serve as a marker of tissue integrity after acute myocardial infarction (Rapacchi et al., 2011). Diffusion tensor imaging (DTI) is the workhorse of cardiac diffusion MRI, and models the diffusion displacement profile as a 3D tensor with a single Gaussian distribution (Basser et al., 1994). It provides information relating to the characterisation of histo-anatomy both in healthy hearts (Gilbert et al., 2012, Healy et al., 2011, Jiang et al., 2004, Poveda et al., 2012) and disease models such as myocardial infarction (Mekkaoui et al., 2012, Strijkers et al., 2009) or hypertrophy (McGill et al., 2012), by assessment of tissue anisotropy and locally prevailling cell orientations, referred to as “fibre orientation”. DTI provides additional indices such as fractional anisotropy (FA), helix angles and transverse angles of fibres. DTI also facilitates 3D fibre tracking and exquisite depictions of the whole-organ prevailing cell orientation, referred to as the cardiac fibre architecture (Sosnovik et al., 2009a), as validated with histology (Cooper et al., 2012, Hsu et al., 1998, Scollan et al., 1998). Importantly, cardiac DTI is well-suited for assessing myocardial microstructure throughout the cardiac cycle in the same heart (Chen et al., 2005, Hales et al., 2012, McGill et al., 2014).

While the single tensor model in DTI has been widely used as an efficient approach for investigating diffusion, Brownian motion of water in tissues is a more complicated process, in no small part due to the presence of multiple populations of cell types and orientations in each imaging voxel. A number of methods have been developed to better reflect multiple fibre populations within a voxel. These include multi-tensor fitting (Hosey et al., 2005), Q-ball imaging (Tuch, 2004), persistent angular structure MRI (Jansons and Alexander, 2003), diffusion orientation transform (Ozarslan et al., 2006), and spherical deconvolution (Tournier et al., 2004). These methods provide an estimate of the radial projections of the diffusion propagator, P (Wedeen et al., 2005) without the single tensor constraint. The development of these methods has been driven by brain imaging, with limited experience in cardiac applications. Therefore, beyond their higher acquisition overhead, the value of these imaging approaches in assessing cardiac microstructure remains to be established.

Another important consideration is that diffusion in tissue is impeded by membranes, organelles and other structures. This leads to diffusion that is hindered and restricted, and gives rise to a diffusion propagator, *P* with a non-Gaussian distribution. If for example, diffusing spins encounter an impermeable restriction during a finite diffusion time, *Δ*, then their measured or apparent diffusion will underestimate their true diffusivity. The cumulant expansion is a useful framework that expresses the magnitude of the diffusion-weighted signal as a power series of the b-value (Kiselev and Il’yasov, 2007). The first and second cumulants describe the Gaussian component of the distribution, while the fourth order cumulant describes the kurtosis, or sharpening, of the peak of *P*. This deviation from Gaussian distribution can be modelled using a number of methods such as diffusion kurtosis imaging (Jensen et al., 2005), hybrid diffusion imaging (Wu and Alexander, 2007), combined hindered and restricted model of diffusion (Assaf et al., 2004), and generalised DTI (Liu et al., 2004). Alternatively, the data can be fitted using gamma-distribution functions (Röding et al., 2012), stretched exponentials (Bennett et al., 2003) or bi-exponential functions (Maier et al., 2004). These methods are more demanding than DTI, requiring acquisition of data with multiple b-values, and they can require a relatively high maximum b-value to provide information about the shape of *P*. This remains a challenge in cardiac imaging, as addressed in a recent initial study (Liu et al., 2011).

Diffusion spectrum imaging (DSI) on the other hand, enables direct reconstruction of *P*, also referred to as the *probability density function* (PDF) (Wedeen et al., 2005). In contrast to other approaches, DSI facilitates quantification of non-Gaussian diffusion and resolution of multiple diffusion orientations in a model-free manner. The PDF can be calculated on a voxel-wise basis as follows:

[1] $${\bar{p}}_{\Delta }\left(r\right)=S_{0}^{-1}{\left(2\pi \right)}^{-3}\int_{{\mathbb{R}}^{3}}|S_{\Delta }\left(q\right)|e^{-iq\cdot r}d^{3}q$$

where ${\bar{p}}_{\Delta }$ is the diffusion spectrum, *S*_0_ is the signal without diffusion weighting, *S*_*Δ*_ is the diffusion-weighted signal, *Δ* is the diffusion separation time, ***r*** is the relative spin displacement, and ***q*** is the diffusion wave vector, where ***q*** = γ**G**δ/2π, and γ is the gyromagnetic ratio, **G** is the diffusion gradient vector and *δ* is the diffusion pulse duration.

Correspondingly, the PDF can be obtained by the Fourier transform of the signal as measured in q-space. Consequently, diffusion-weighted data are sampled on a 3D Cartesian grid in q-space by acquiring data with different diffusion gradient strengths and orientations. For efficiency and isotropic resolution, the sampling scheme is usually restricted to a sphere and avoids sampling the corners of q-space. The PDF can then be integrated radially to yield the *orientation distribution function*, which describes its orientation with no prescribed limitation on the number of dominant directions. Likewise, the shape of the PDF can be described by parameters such as the probability at zero distribution, the full-width-half-maximum and its kurtosis (Lätt et al., 2008). Further fitting of the PDF to various non-Gaussian models is possible, providing access to yet more parameters. Due to the heavy acquisition burden, DSI in the heart is presently restricted to *ex vivo* samples (Sosnovik et al., 2009b, Teh et al., 2014a, Wang et al., 2010).

Despite the wealth of information provided by diffusion MRI, its application in clinical cardiac imaging remains limited. *In vivo* diffusion MRI is challenging primarily as the scale of motion is usually much greater than that of the measured water diffusion. Appropriate strategies must thus be adopted to compensate for the effect of cardiac and respiratory motion (Dou et al., 2002, Nguyen et al., 2013, Tseng et al., 1999). As the contrast in diffusion imaging is dependent on signal attenuation, it is intrinsically signal-to-noise ratio (SNR) limited. This increases the number of averages needed, leading to longer scan times and lower resolution. The latter can give rise to partial volume effects, especially in the apical region, and makes it difficult to delineate complex structures. Finally, the gradient strength available on standard clinical MRI systems limits the achievable b-value.

Cardiac diffusion MRI in small animals adds further challenges, due to the high heart rates – typically around 400 to 600 beats per minute in rats and mice – and the orders of magnitude smaller imaging volumes. These, in turn, lead to greater difficulties in motion compensation and lower SNR, respectively. While the higher field strengths used in small animal imaging help to ameliorate the SNR decrease, they also lead to longer *T*_1_ and shorter *T*_2_ relaxation times that reduce SNR efficiency. Conversely, the increased gradient strength available on many preclinical systems helps to minimise echo times and mitigate *T*_2_ relaxation, while extending the range of b-values that can be acquired. As *ex vivo* diffusion MRI is not constrained by respiratory and cardiac motion, 3D high-resolution isotropic scans can be acquired with high SNR, albeit at the cost of increased scan time. Due primarily to motion sensitivity, the majority of the literature reports data from *ex vivo* fixed samples, with few exceptions obtained *in vivo* (Sosnovik et al., 2014).

As a bridging step, several groups including ours have developed protocols to perform DTI in the living, isolated and arrested heart, thus mitigating motion artifacts (Chen et al., 2005, Garrido et al., 1994, Hales et al., 2012, Hsu et al., 2001, Kohler et al., 2003, Scollan et al., 1998). *Ex vivo* perfusion of the living heart is a well-established and important model in basic cardiovascular research, as it affords better control over manipulation and observation, compared to the *in vivo* setting. The Langendorff-perfused heart (Langendorff, 1897) in particular represents an interesting intermediate step between the *in vivo* beating heart and the *ex vivo* fixed organ, as hearts can be arrested, paced, or allowed to beat freely in the absence of respiratory motion (French, 2012), while maintaining near-physiological conditions of the living myocardium (Bell et al., 2011). Arresting the heart avoids the issue of cardiac contraction-induced deformation, and helps to preserve energy. This model has been used extensively for the study of cardiac metabolism using magnetic resonance spectroscopy (Allis et al., 1989, Bailey et al., 1981, Jacobus et al., 1977, Malloy et al., 1990, Ugurbil et al., 1984), but also for the development and validation of new cardiovascular MRI techniques such as first-pass cardiac perfusion (Atkinson et al., 1990), flow measurements (Burstein, 1991), and myocardial arterial spin labeling (Williams et al., 1993). The improved control over motion is especially appealing in applications where bulk motion represents a major obstacle, such as in cardiac DTI (Chen et al., 2005, Garrido et al., 1994, Hales et al., 2012, Hsu et al., 2001, Kohler et al., 2003, Scollan et al., 1998).

Characterising dynamic changes in the micro-architecture relies on observations of cardiac deformation during the contractile cycle. Such observations shed light on cardiac mechanics and structure-function relationships. They also facilitate the development and validation of conceptual and computational models of the heart (Gilliam and Epstein, 2012, Helm et al., 2005, Rudy et al., 2008, Vadakkumpadan et al., 2010, Young and Frangi, 2009). This information cannot be obtained from imaging chemically fixed hearts. Recently, Hales et al. used a custom-made perfusion rig to non-invasively assess myocardial fibre architecture in the same heart in two different mechanical states – slack and contracture – in a horizontal magnet (Hales et al., 2012). In their design, the MRI setup was completely decoupled from the perfusion rig, employing a radio-frequency (RF) coil outside the sealed chamber containing the heart. This configuration limited MRI sensitivity, which then required multiple averages and long scan times to achieve adequate spatial resolution and SNR. Moreover, hearts were perfused at room temperature (21 °C) using a gravity-fed system. To mimic more physiological conditions, it is desirable to (i) maintain mammalian hearts at body temperature, as cardiac electrophysiology and mechanics are highly temperature sensitive, and (ii) have control over the flow rate, both to sustain metabolic demand, and to avoid changes in coronary vascular load. To this end, hearts can be fully immersed in heated buffer, and/or pump-perfused with heated buffer and enclosed in a water-tight chamber (Bell et al., 2011). In both cases, ingress of air bubbles, asymmetric coil loading and susceptibility artifacts need to be considered.

The present study aims to (i) characterise the microstructure in rat hearts in multiple (here three) deformation states using diffusion MRI, (ii) demonstrate how specialised hardware can improve the sensitivity of measurements and support more physiological conditions and (iii) show how a more comprehensive description of the diffusion profile expands our ability to characterise the microstructural properties of the heart.

Refinement of the RF coil technology can improve the SNR of the MRI experiments, leading to significant differences in the accuracy and precision of the measured DTI parameters (Giannelli et al., 2011, Lohezic et al., 2014). Here, we introduce a novel perfusion rig that integrates the RF coil within the organ perfusion chamber. This increases the fraction of the coil's sensitive volume being taken up by the sample, or the *filling factor*, and therefore the SNR. Further optimisation to the previous design include (i) improved temperature control to allow for experiments at body temperature, (ii) constant flow perfusion, and (iii) integration of a pressure-sensing balloon in the left ventricle (LV) cavity to assess and/or control the mechanical state of the heart. DTI experiments were performed to characterise cardiac microstructure in multiple mechanical states in the same heart. SNR, *T*_1_ and *T*_2_ were measured to quantify the utility of the integrated RF coil and assess sample stability. Subsequently, samples were fixed in slack or contractured states and scanned with isotropic 3D DSI to additionally investigate non-Gaussian diffusion changes characteristic of the different mechanical states.

## 2 Methods

### 2.1 Hardware

#### 2.1.1 MRI system and RF coils

All imaging was performed using a horizontal 9.4T (400 MHz) MRI system (Agilent Technologies, Santa Clara, USA), bore size = 210 mm, a VNMRS Direct Drive console, and a shielded gradient system (maximum gradient strength = 1 T m^−1^, rise time = 130 μs, and inner diameter = 60 mm). A dedicated fixed tune-and-match birdcage coil (inner diameter = 20 mm, length = 25 mm; Rapid Biomedical, Rimpar, Germany) was used for transmit/receive in the perfused heart experiments. For 3D DSI of *ex vivo* fixed hearts, a different transmit/receive birdcage coil was used (inner diameter = 20 mm; Rapid Biomedical, Rimpar, Germany), where tune-and-match were adjusted to each sample.

#### 2.1.2 Perfusion setup

**Fig. 1:**
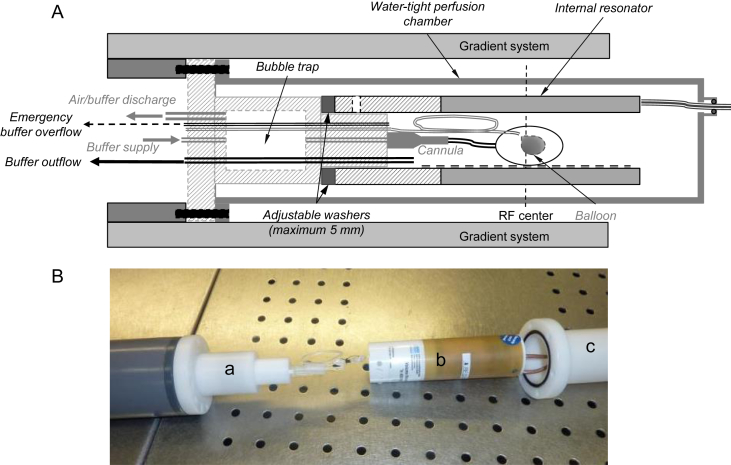
Optimised setup for magnetic resonance imaging of the perfused rat heart. A: Design of the perfusion setup. The transmit/receive resonator was integrated inside the perfusion chamber to improve the filling factor and thus the SNR. A bubble trap was built into the perfusion head to prevent air bubbles from reaching the heart, and a balloon was added to the system for improved control over the state of contraction. B: Photograph of the perfusion setup prior to assembly. The heart is first connected to the cannula and the balloon then inserted in the left ventricle. The perfusion head (a), internal resonator (b) and perfusion chamber (c) are then assembled in a concentric fashion.

The setup presented by Hales et al. (Hales et al., 2012) was modified by integrating the RF coil inside the perfusion rig (Lohezic et al., 2014), as close as possible around the heart (Fig. 1), and by delivering oxygen-enriched, albumin-containing perfusate under constant flow and at body temperature to the heart inside the magnet. Providing heated and oxygenated perfusate to the heart is confounded by heat loss that occurs along the perfusion line. This alters the solubility of gases in solution and can give rise to the formation of bubbles. To avoid ingress of bubbles into the heart, a bubble trap was built into the perfusion head. Additional active perfusate/air discharge allowed rapid turnover of the solution and improved temperature stability. The physiological solution was first preheated to body temperature in water-jacketed reservoirs and saturated with oxygen by bubbling. The perfusate was then delivered to the heart in the MRI scanner bore via water-jacketed tubing using a pulsation-free piezo micro-pump cascade (Bartels Mikrotechnik GmbH, Dortmund, Germany) at a constant flow rate of 5 mL min^−1^. A final modification to the setup included the provision of a line to a balloon, made of thin plastic film, inside the LV cavity. Inflation of the balloon was controlled manually, from outside the magnet room, using a 1 mL syringe. Pressure sensors were integrated in the perfusion and balloon lines, to monitor perfusion and LV pressures, respectively.

### 2.2 Sample preparation

#### 2.2.1 Perfused rat heart experiments

All animal work was conducted in accordance with the UK Home Office Guidance on the Operation of Animals (Scientific Procedures) Act of 1986, and was approved by the University of Oxford's ethical review board. Hearts were excised from seven female Sprague–Dawley rats (body weight = 256 ± 13 g) after cervical dislocation, and connected to the perfusion setup via swift cannulation of the aorta for coronary perfusion in Langendorff mode. An incision was made into the pulmonary artery wall distal to the right ventricular outflow valve to prevent intra-ventricular fluid accumulation. After the initial wash using normal Tyrode solution (in [mM]: NaCl 140.0; KCl 5.4; MgCl_2_ 1.0; CaCl_2_ 1.8; HEPES 5.0; Glucose 11.0), the heart was cardioplegically arrested using a modified high-K^+^ Tyrode solution (in [mM]: NaCl 105.0; KCl 25.0; MgCl_2_ 1.0; HEPES 10.0; Glucose 11.0; BDM 10.0; 20 μM Blebbistatin and 1.5% albumin). All solutions were titrated to pH 7.4. A balloon was inserted into the LV cavity via an incision in the left atrium and through the atrio-ventricular valve. Contracture was induced in four hearts by switching the perfusate to Na^+^-free Li^+^-Tyrode solution (in [mM]: LiCl 125.0; KCl 5; MgCl_2_ 1.0; CaCl_2_ 2.5; HEPES 10.0; Glucose 11.0).

#### 2.2.2 Fixed hearts

Following the perfused live heart experiments, two samples were chemically fixed in contracture. Fixation was via coronary perfusion with iso-osmotic Karnovsky's fixative, containing 0.45% paraformaldehyde, 0.57% glutaraldehyde, and 0.97% sodium cacodylate (Electron Microscopy Sciences, Hatfield, USA). For comparison, two additional hearts were excised and fixed in their cardioplegically arrested slack state via coronary perfusion with Karnovsky's fixative (Solmedia, Shrewsbury, UK). The four fixed hearts were stored in fixative for at least 24 h at 4 °C, rinsed in iso-osmotic cacodylate buffer and embedded in iso-osmotic 2% low-temperature melting agar in 20 mm glass tubes.

### 2.3 Setup validation

#### 2.3.1 Sample stability at 37 °C

Potential progression of myocardial oedema over time was investigated in three rat hearts (*N* = 3) by measuring stability of *T*_1_ and *T*_2_ over a period of 170 min. The time delay between excision and the start of imaging was about 35 min. During the imaging period, myocardial *T*_1_ and *T*_2_ values and LV wall thickness were measured 10 times in 17 min cycles. *T*_1_ was assessed using a 2D multi-slice spoiled gradient echo sequence with inversion recovery: repetition time (TR) = 5 ms, echo time (TE) = 1.3 ms, 40 images with inversion times ranging from 80 to 6320 ms, flip angle = 8°, field-of-view (FOV) = 20 × 20 mm, matrix = 128 × 128, slice thickness = 1 mm, slices = 3, averages = 2. *T*_2_ was measured using a 2D multi-slice multi-echo spin echo sequence: TR = 2.5 s, 4.6 ≤ TE ≤ 73.3 ms, echo train length = 16, FOV = 20 × 20 mm, matrix = 128 × 128, slice thickness = 1 mm, slices = 5, averages = 2. *T*_1_ and *T*_2_ values were calculated on a voxel-by-voxel basis by applying a mono-exponential fit to the magnitude images using purpose-built software written in IDL (Exelis, McLean, VA, USA). The myocardium was then manually segmented at each time point and mean values computed across the myocardium. The LV wall thickness was measured at two locations in a mid-ventricular slice from the *T*_2_ experiments using VnmrJ 3.2 (Agilent Technologies, Santa Clara, USA). A two-sided, paired *t*-test was used to compare the parameters from the first and last time points. *P* ≤ 0.05 was used throughout the study to indicate a significant difference between means.

#### 2.3.2 SNR measurements

SNR was assessed in the B_0_ images of the diffusion MRI data acquired in the slack state. The signal was measured on a voxel-by-voxel basis as the median value over a 3 × 3 kernel. Noise was assessed in each slice by computing the standard deviation in a manually drawn region-of-interest (ROI) in the background. SNR maps were then obtained in the manually segmented myocardium by dividing the measured signal by the noise in each respective slice. Four datasets from a previous study using an external resonator (Hales et al., 2012) were also retrospectively analysed in the same way. Values were corrected for different voxel sizes and temperatures:

[2] $${\text{SNR}}_{\text{corrected}}=0.9739\times \text{SNR}\times \frac{V_{C}}{V_{P}}$$

where the factor 0.9739 corrects for the temperature difference (i.e. 37 °C for the current experiments *versus* 21 °C in the previous study), and *V*_*P*_ and *V*_*C*_ are the voxel sizes in the previous and current experiments, respectively (Callaghan, 1993). A two-sided, unpaired *t*-test was used to compare the SNR from the two series of experiments.

### 2.4 DTI of living isolated hearts in three mechanical states

#### 2.4.1 DTI acquisition

Hearts (*n* = 4) were scanned using a diffusion-weighted multi-shot fast spin echo sequence. Images were acquired in the short-axis orientation of the heart in 13 contiguous slices covering the heart from apex to mitral valve using the following parameters: TR/TE_eff_ = 1000/15 ms, echo train length = 8 (central k-space acquired at the first echo), FOV = 20 × 20 mm, matrix = 128 × 128, in-plane resolution = (156 μm)^2^, slice thickness = 1 mm, averages = 12, acquisition time = 35 min per state. Unipolar diffusion sensitizing gradients were used with maximum diffusion gradient strength, *G*_max_ = 310 mT m^−1^, gradient duration, *δ* = 2.5 ms, separation time, *Δ* = 9.6 ms, and maximum *b*-value, *b*_max_ = 508 s mm^−2^ including contribution from imaging gradients and cross-terms. Ten non-collinear gradient directions were used (Jones et al., 1999).

#### 2.4.2 Multiple mechanical states

Four rat hearts were subjected to DTI with the balloon at three different inflation levels. First, the balloon was inflated just enough to be in contact with the LV wall in cardioplegic arrest (slack state). The balloon was then inflated to a pressure of 70 mmHg (volume loading). Finally, the balloon was deflated, and ventricular contracture was induced by switching the perfusate to Na^+^-free Li^+^-Tyrode solution. The third DTI scan was performed 20 min after switching solutions to ensure complete tissue equilibration. The orientation of the acquisition plane was adjusted for each mechanical state to correspond to the short axis of the heart.

#### 2.4.3 DTI analysis

Diffusion tensors were calculated on a voxel-by-voxel basis via a weighted linear least-squares fitting method, using in-house software developed in IDL. Tensors were diagonalised to obtain the eigenvectors (*υ*_1_, *υ*_2_, *υ*_3_) and corresponding eigenvalues (*λ*_1_, *λ*_2_, *λ*_3_). From these, maps of FA and ADC were calculated using established methods (Basser et al., 1994). Same-organ measurements were compared using a repeated measures analysis of variance. When the significance threshold of *P* ≤ 0.05 was reached, a Tukey test was subsequently performed to find means that were significantly different from each other. Myocardial fibre orientation was expressed in terms of the helix angle *α* (Streeter et al., 1969), defined as the angle between (a) the projection of *υ*_*1*_ onto the circumferential–longitudinal plane (tangential plane parallel to the epicardial surface) and (b) the circumferential-radial (or chamber-horizontal) plane. For that purpose, the long axis of the LV was determined in each mechanical state by manually selecting the centre of the LV cavity in several slices. The long axis was then calculated by linear regression of these points, and the chamber–horizontal plane defined as perpendicular to this axis. Helix angles were defined as negative and positive for left- and right-handed helical orientations, respectively. The voxels were then binned according to the measured helix angle into left-handed fibres (LHF: *α* ≤ −30°), circumferential fibres (CF: −30° < *α* < 30°) and right-handed fibres (RHF: *α* ≥ 30°). The papillary muscles and trabeculae were excluded from the analysis. The changes in the partition of fibres between the three mechanical states were analysed using a 3 × 3 chi-square test.

### 2.5 DSI of *ex vivo* hearts fixed in two mechanical states

#### 2.5.1 DSI acquisition

High resolution isotropic DSI data were acquired with a 3D spin echo echo planar imaging sequence with: TR/TE = 1000/18 ms, echo train length = 20, FOV = 24 × 20 × 20 mm, resolution = (250 μm)^3^, total acquisition time = 47 h. Forward and reversed readout polarity data were acquired to correct for small inconsistencies between odd and even lines of *k*-space (van der Zwaag et al., 2009). Unipolar diffusion sensitizing gradients were used with *G*_max_ = 846 mT m^−1^, *δ* = 5 ms, *Δ* = 10 ms, and *b*_max_ = 10,000 s mm^−2^. Q-space was sampled in 257 steps in a 3D grid circumscribed by half a sphere, taking advantage of symmetry in q-space to halve the acquisition time. To improve accuracy in q-space sampling, the gradient strengths were adjusted for each diffusion step, based on a previous calibration scan using an isotropic phantom (Teh et al., 2014b).

#### 2.5.2 DSI analysis

A 3D Fourier transform was applied to the signal in a voxel-by-voxel manner in q-space to generate the PDF (Equation (1)). Maps of mean squared length (MSL) (Kuo et al., 2008), generalised fractional anisotropy (GFA) (Tuch, 2004) and mean kurtosis (MK) (Lätt et al., 2008) were calculated within the myocardium, and normalised histograms plotted using in-house software developed in Matlab (Mathworks, Natick, USA). Summary data over all voxels in the myocardium of the two slack hearts were compared to that in the two contractured hearts. Fibre tracts were generated with Diffusion Toolkit and displayed in Trackvis (Wang et al., 2007). Tracts were seeded (i) by growing spherical ROIs centered in the LV cavity by linearly stepping their radii between 4 and 24 voxels as normalised by the average wall thickness in a mid-ventricular short-axis slice, and (ii) from single voxel radius spherical ROIs located across the septal and lateral walls of the LV. The tracking utilised a modified FACT algorithm (Mori et al., 1999) that allowed for multiple intravoxel diffusion orientations. It was constrained by an FA mask ranging from 0.08 to 1, and the angular-deviation threshold was 30°.

## 3 Results

### 3.1 Setup validation

#### 3.1.1 Sample stability

The three hearts scanned in the initial control experiments showed little sign of gross anatomical change over the nearly three-hour period of investigation, and no significant changes in the mean *T*_1_, *T*_2_ or LV wall thickness were detected. The mean myocardial *T*_1_ values were 2.03 ± 0.13 s (*t* = 0) and 2.16 ± 0.01 s (*t* = 170 min; *P* = 0.26). The mean myocardial *T*_2_ values were 39.4 ± 6.6 ms (*t* = 0) and 38.8 ± 5.3 ms (*t* = 170 min; *P* = 0.64). LV wall thickness values were 1.37 ± 0.02 mm (*t* = 0) and 1.39 ± 0.04 mm (*t* = 170 min; *P* = 0.55).

#### 3.1.2 SNR measurements

**Fig. 2:**
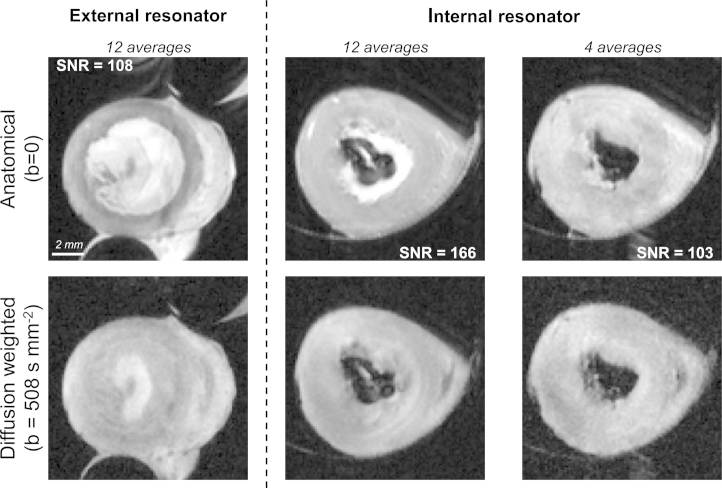
SNR improved with the integration of the RF coil inside the perfusion chamber. Typical images obtained using a previous perfusion setup where the resonator was placed outside the perfusion chamber (left) and the proposed design (right). The images are displayed at the same scale.

The RF coil filling factor for our configuration compared favourably to that from the previous study (0.25 vs 0.047). This contributed to a 75% increase in mean SNR in the B_0_ images (168 ± 25 vs 96 ± 5; *N* = 4; *P* = 0.001). Representative images obtained with either setup are shown in Fig. 2.

### 3.2 DTI of living isolated hearts in three mechanical states

**Fig. 3:**
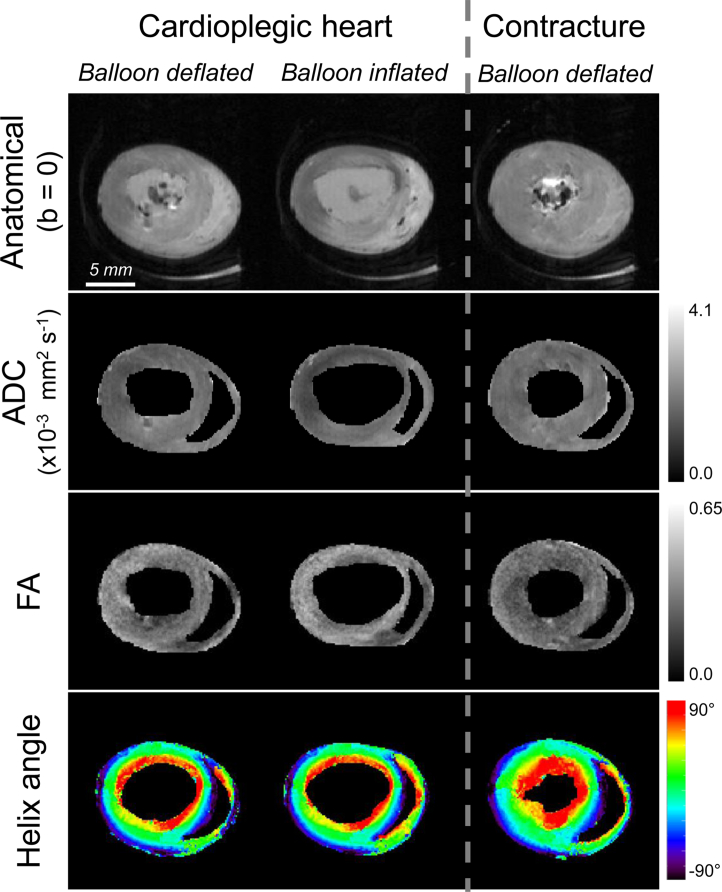
Anatomical images and DTI maps in three mechanical states obtained in the same perfused rat heart. Apparent diffusion coefficient (ADC), fractional anisotropy (FA) and helix angle maps from the live isolated rat heart in slack (left), in volume overload conditions (middle) and after induction of contracture (right). The images and maps are displayed at the same scale. Note: slices are not exactly matching due to different orientations of states.

Images and DTI parameter maps obtained from the 3-state protocol are shown in Fig. 3, where the slack state (cardioplegic solution, balloon deflated) is taken to roughly represent diastole (left), the balloon-inflated state models increased venous return (middle), and the lithium-induced contracture mimics end-systole (right).

The mean and standard deviation of the DTI measurements over the whole myocardium are listed in Table 1. When averaged over the myocardial volume, statistically significant differences between the three states were found in FA (*P* = 0.001), but not in ADC (*P* = 0.46). The post-hoc Tukey test found significant differences in FA between slack state and contracture (*P* < 0.01, mean difference: 0.04) and between the volume overload and contracture (*P* < 0.01, mean difference: 0.05). None of the eigenvalues showed statistically significant changes between the three states (*P* > 0.3). On the other hand, the changes in the ratios *λ*_1_/*λ*_2_ and *λ*_2_/*λ*_3_ were statistically significant (*P* = 0.016 and 0.001, respectively). The post-hoc Tukey test showed significant differences in *λ*_1_/*λ*_2_ between the volume overload and contracture (*P* < 0.05, mean difference: 0.09) and in *λ*_2_/*λ*_3_ between slack state and contracture (*P* < 0.01, mean difference: 0.09) and between the volume overload and contracture (*P* < 0.01, mean difference: 0.11).

**Table 1:** Mean and standard deviation (SD) of DTI parameters (ADC: apparent diffusion coefficient; FA: fractional anisotropy; *λ*_1_, *λ*_2_ and *λ*_3_: 1st, 2nd and 3rd eigenvalues) over the myocardial volume in three mechanical states.

	Slack		Overloaded		Contracture	
	Mean	SD	Mean	SD	Mean	SD
ADC (×10^−3^ mm^2^ s^−1^)	1.70	0.10	1.56	0.26	1.62	0.21
FA	0.32	0.02	0.32	0.02	0.28	0.02
*λ*_1_ (×10^−3^ mm^2^ s^−1^)	2.31	0.14	2.11	0.37	2.08	0.31
*λ*_2_ (×10^−3^ mm^2^ s^−1^)	1.69	0.09	1.53	0.26	1.58	0.23
*λ*_3_ (×10^−3^ mm^2^ s^−1^)	1.21	0.09	1.10	0.22	1.20	0.18
*λ*_1_/*λ*_2_	1.40	0.04	1.41	0.05	1.35	0.02
*λ*_2_/*λ*_3_	1.46	0.06	1.47	0.07	1.35	0.05

**Fig. 4:**
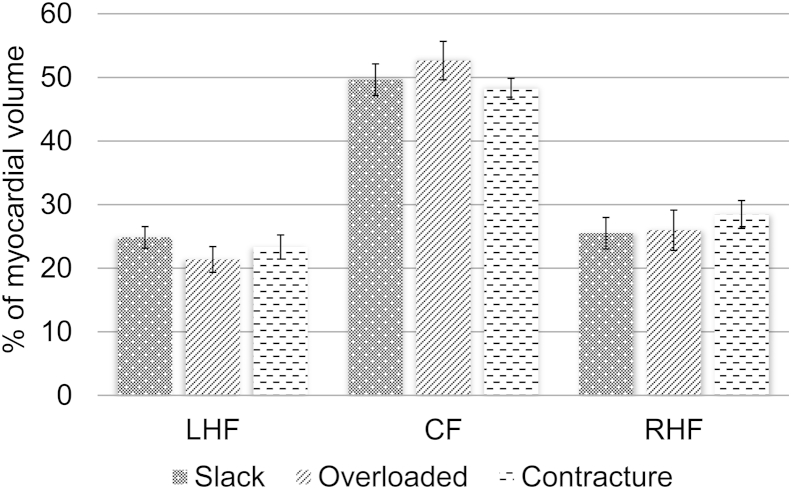
Summary of changes in helix angles in multiple mechanical states. Mean proportions of left-handed fibres (LHF: *α ≤* −30°), circumferential fibres (CF: −30° < *α* < 30°) and right-handed fibres (RHF: *α* ≥ 30°) observed across the 4 perfused hearts. The changes in the partition of fibres between the three mechanical states were statistically significant (Chi-square: *P* < 10^−4^).

The changes in helix angles between the three mechanical states are shown in Fig. 4. The proportion of LHF decreased from 24.9% to 21.4% when the balloon was inflated (slack to volume overload), while that of RHF increased from 25.5% to 26.0%. The fraction of CF increased from 49.6% to 52.6%. Conversely, contracture gave rise to a decrease in the proportion of LHF from 24.9% to 23.3% (slack to contracture), while the proportion of RHF increased from 25.5% to 28.4%. While the amplitudes of changes were relatively modest, chi-square analysis showed that they were statistically significant (*P* < 10^−4^).

### 3.3 DSI of *ex vivo* hearts fixed in two mechanical states

**Fig. 5:**
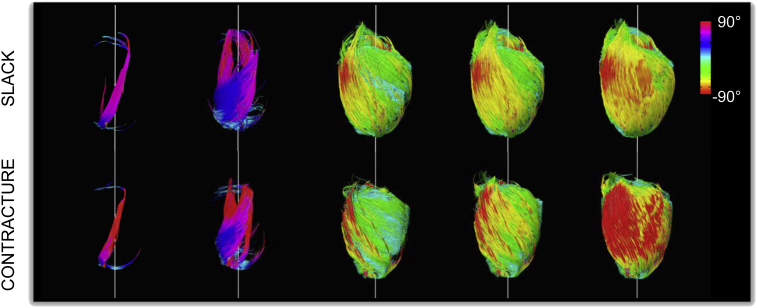
3D fibre tracking in hearts in slack (top) and contractured (bottom) states shows progression from right to left helical fibres, moving from the subendocardium to the subepicardium. Tracts were progressively seeded with a spherical ROI of increasing radius, normalised to wall thickness and centered in the left ventricular cavity. Tracts were colour coded by helix angle where negative and positive angles correspond to left handed and right handed fibres, respectively.

By progressively enlarging the ROIs used for seeding tracts, we observed a shift in fibre orientation from the almost longitudinal fibres of the trabeculae, through the right helical fibres of the subendocardium, circumferential fibres of the mid-myocardium and left helical fibres of the subepicardium (Fig. 5). This principal distribution was preserved in both slack and contractured states.

**Fig. 6:**
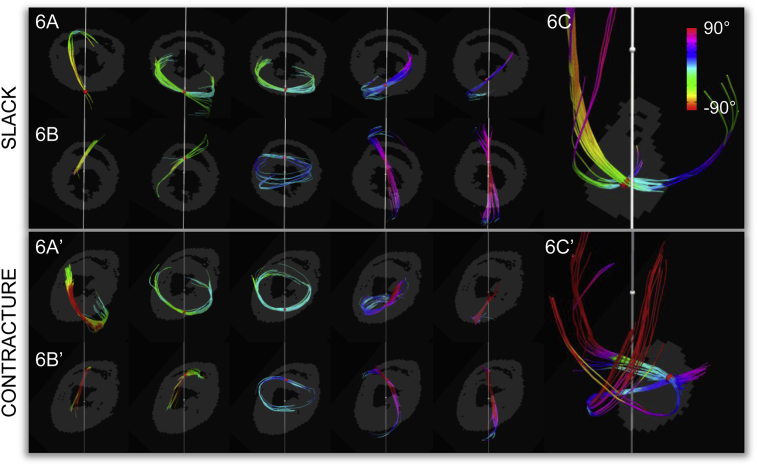
Region-of-interest (ROI) based fibre tracking illustrates transition from subepicardial (left) to subendocardial (right) fibres in the lateral wall of the left ventricle (Figs. 6A & A′) and the septal wall of the left ventricle (Figs. 6B & B′). Tracts were seeded with a single voxel radius ROI in a mid-ventricular short-axis slice, and colour coded by helix angle. These are overlaid on single slice masks. Fibre tracking in an apical slice reflects the presence of regions of multiple predominant cell orientations (Figs. 6C & C′). It is important not to confuse this tracking of locally prevailing cell orientations with physically continuous heart muscle strands as cardiomyocytes are discrete and only about 200 μm long.

Tracts seeded in single voxel radius ROIs in the septal and lateral LV walls further illustrate the transition between left helical fibres with negative helix angles in the subepicardium to right helical fibres with positive helix angles in the subendocardium (Fig. 6). Inspection of the tracts in the LV lateral walls show that tracts in slack hearts form a half spiral and retain relatively uniform helix angles throughout their progression (Fig. 6A), while tracts in contractured hearts have helix angles that vary more (Fig. 6A′), in line with torsional effects during mechanical activation. Tracts seeded near the apex show regions of crossing fibres (Fig. 6C and C′). These may reflect the apical crossing of the longitudinal deep myocardial strands with the oblique superficial strands (Ho, 2009). In the slack state, these tracts have a smooth ellipsoidal profile, whereas in the contractured heart they are flattened in the long-axis, and may indicate a change in fibre geometry associated with longitudinal shortening during systole.

**Fig. 7:**
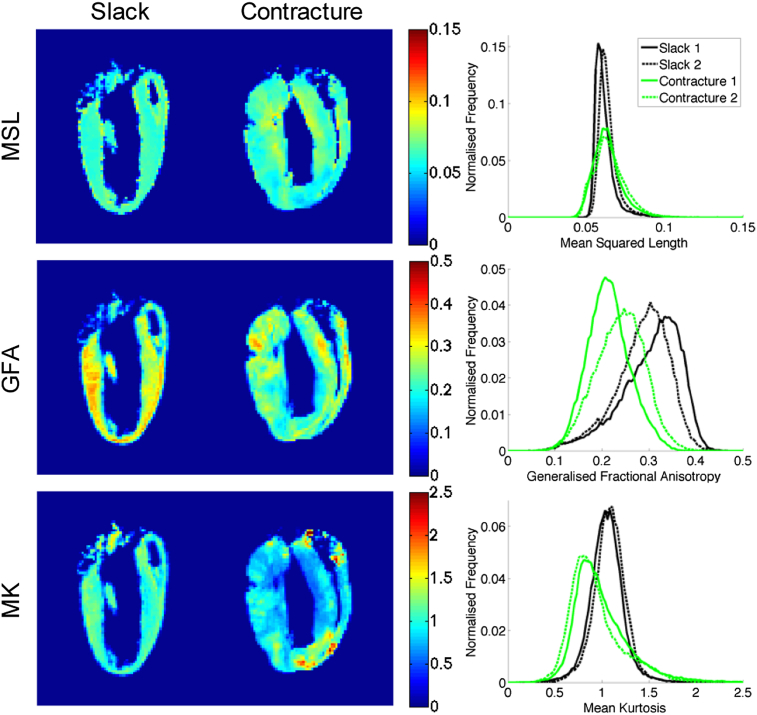
DSI parameter maps including mean squared length (MSL) (top), generalised fractional anisotropy (GFA) (middle) and mean kurtosis (MK) (bottom). Mid-sagittal long-axis views of *ex vivo* rat hearts in slack (left) and contractured (middle) states are presented, alongside normalised histograms from 3D whole heart data (right) from 2 hearts each (solid and dashed lines) in slack state (black) and in contracture (green). Hearts in contracture show evidence of decreased GFA and MK as well as larger variation in MSL and MK.

Comparison of DSI parameters over the whole heart reveals a number of striking differences (Fig. 7). Relative to hearts in the slack state, contracture is associated with lower GFA and MK, as can be seen from the leftward shift in the normalised histograms. The maps also appear more heterogeneous, with greater standard deviations in the MSL and MK. The values are summarised in Table 2.

**Table 2:** Summary DSI data comparing 3D whole heart data from slack and contractured hearts. The following were observed in the contractured vs slack hearts: (i) mean squared length (MSL) showed a substantial increase in standard deviation, (ii) generalised fractional anisotropy (GFA) values decreased along with its standard deviation, and (iii) mean kurtosis (MK) decreased slightly while its standard deviation increased dramatically.

	Slack		Contracture	
	Mean	SD	Mean	SD
MSL	0.0627	0.0063	0.0643 (+2.6%)	0.0095 (+51%)
GFA	0.294	0.061	0.227 (−23%)	0.050 (−18%)
MK	1.07	0.17	0.970 (−9.3%)	0.295 (+74%)

## 4 Discussion

### 4.1 Setup validation

The aim of the study was to develop and test an optimised MRI-compatible Langendorff perfusion setup for examination of live isolated rat hearts. To this end, we first integrated the RF coil into the perfusion chamber around the heart, thus improving the coil filling factor and resultant SNR. Considering only the increased filling factor, one would expect an improved transmit efficiency or receive sensitivity of $\sqrt{25\%/4.4\%}\approx 230\%$. In fact, the SNR gain was 75%. The lower increase could be due to the lower available unloaded quality factor of RF coils with small geometric dimensions. The SNR gain can be invested in reducing scan time, increasing the spatial resolution and/or increasing the number of diffusion directions used. Higher SNR improves the precision and accuracy of DTI estimates in fixed rat hearts (Hales et al., 2010, Lohezic et al., 2014). This could be used to reduce the number of animals needed for studies. Besides DTI, higher SNR would enhance the prospects of other SNR-constrained MRI techniques, including cardiac arterial spin labeling for the assessment of the myocardial perfusion (Bauer et al., 2001) and proton spectroscopy (Schneider et al., 2000).

The insertion of a balloon in the LV provided improved control over cardiac contraction. The general approach was similar to Chen et al. (2005), who used a balloon inserted in the LV cavity to explore isovolumic contraction in early systole. However, in that case, only two mechanical states were imaged in the same heart and one of them after fixation. In future work, the balloon will be used in association with a pressure feedback controller, which may enable examination of paced or freely beating hearts. The resulting pressure signal could be used to synchronize the MRI acquisition with the cardiac mechanical cycle (Schneider et al., 2000) and to simultaneously characterise cardiac function (Spindler et al., 2002). As all electronic equipment including pumps, heater, and pressure probes were located outside the Faraday cage, the perfusion chamber was fully MRI-compatible and did not interfere with the acquisitions.

Balloon insertion increased preparation time from ∼15 min to ∼35 min to the start of MRI acquisition. However, there were no signs of progressive oedema or other gross anatomical changes during the study period of nearly three hours. We attribute this to the use of body temperature and albumin-enriched buffer, which provided more physiological experimental conditions. Also, we have prevoiously shown that the largest step-change in cardiac tissue volume occurs very soon after cardiac excision, prior to the earliest time points at which MRI data can be obtained even if no balloon is inserted (Bub et al., 2010). This is probably due to a combination of changes in transmural pressure gradients and exposure to saline. Live biological samples remained stable for 170 min from the start of measurements, well beyond the 120 min duration of the three-state DTI protocol. This leaves open the option to extend the scan in order to increase either the spatial resolution or the number of diffusion directions.

### 4.2 DTI of living isolated hearts in three mechanical states

Diffusion MRI is inherently sensitive to motion-induced phase errors, which lead to errors in quantification. This was avoided in our study by the use of isolated and arrested hearts, eliminating the need for physiological gating. Moreover, this enabled the use of a multi-shot acquisition technique, as phase errors arising from motion between shots are negligible. This increased the maximum achievable resolution compared to single shot methods, due to the shorter echo times and reduced T_2_ blurring. The use of fast spin echo acquisition was robust against susceptibility artifacts (Bastin and Le Roux, 2002) that could arise from incomplete filling of the perfusion chamber.

The changes observed in the partition of myocardial fibres into LHF, CF and RHF between slack and contractured hearts were consistent with a previous study (Hales et al., 2012). We also observed small but significant changes in the partition of myocardial fibres when comparing the volume overload state to the other states. As expected with the higher experimental temperature, the values of ADC were notably higher than in a previous report (Hales et al., 2012). Furthermore, the changes in FA and the ratios *λ*_1_/*λ*_2_ and *λ*_2_/*λ*_3_ between the three mechanical states were found to be significant, which was not the case in the previous study. The gain in SNR obtained by integrating the RF coil inside the perfusion chamber results in improved accuracy and precision of the DTI estimates (Lohezic et al., 2014), which could explain the statistically significant changes.

In the present study, only the changes in the orientation of the primary eigenvector were investigated. Analysis of the 2nd and 3rd eigenvectors may additionally yield information on the so-called myocardial sheet orientations (laterally-enforced layers of myocardial cells), which have been shown to contribute to ventricular wall deformation during cardiac contraction (Hales et al., 2012). This will be explored in more detail in future research.

Establishing reference ranges of ADC, FA and fibre orientations in the healthy heart is key to identifying changes due to disease and for multi-centre comparison of data. Current reports of ADC and FA measured *in vivo* vary widely, largely stemming from the different acquisition methods used which result in dissimilar sensitivities to motion. Potential factors leading to such variation include the choice of MRI pulse sequence, motion compensation of the diffusion gradients, and cardiac and respiratory gating, if used. As methods in mitigating and compensating for motion continue to improve, imaging of the isolated live heart can serve as a useful benchmark for the establishment of reference values, and be used for validating new methods in the absence of motion, prior to *in vivo* application. At the same time, it is important to recognize the contribution of partial volume effects, which manifest ubiquitously given the large voxel sizes with respect to the cardiac microstructure. An informed assessment of the limitations of the models used to fit the data is thus needed. For instance, in regions containing multiple fibres, the tensor model leads to ambiguity in the origin of FA changes (Wheeler-Kingshott and Cercignani, 2009).

### 4.3 DSI of *ex vivo* hearts fixed in two mechanical states

3D whole heart tractography revealed the characteristic transmural transition of helical fibre orientations, as established using histological methods (Greenbaum et al., 1981, Streeter et al., 1969). Apical regions were found to contain voxels with multiple fibre tracts, underscoring the utility of DSI in reconstructing fibre orientations in regions with complex local cell arrangements. It is worthwhile to note that (i) fibres as referred to in the text, describe a cluster of cardiomyocytes characterised by a slowly varying predominant diffusion direction, and are not long, continuous muscle strands *per se*; and (ii) the diffusion tractography performed here is a simplification that does not reflect the complex branching patterns that occur between cardiomyocytes that are arranged in complex 3D structural domains, including sheets. Unlike histology, DSI can be used to study the intact cardiac architecture isotropically in 3D without distorting tissue and in a non-destructive manner. However, as with many other forms of high-resolution *ex vivo* imaging, it involves prior chemical fixation, which gives rise to changes in tissue geometry, membrane permeability, and ADC (Sun et al., 2003). These changes are dependent on the fixative used, and we opted for an iso-osmotic version of Karnovsky's to reduce tissue shrinkage and deformation (Hales et al., 2011, Burton et al., in press).

DSI also facilitated investigation of changes in cardiac microstructure in response to contraction. For example, the reduction in GFA that was observed could be explained by the shortening of fibres along their length and radial broadening, which would have led to reduced anisotropy. Such interpretation may be ambiguous in regions of crossing fibres using FA measures and diffusion models that are limited to a single primary orientation (Wheeler-Kingshott and Cercignani, 2009), but neither DSI nor GFA are thus constrained. MK and MSL maps had more heterogeneous distributions across the myocardium, and regions with higher MK generally corresponded to regions of lower MSL, and *vice versa*. It is reasonable to postulate that lower MK values describe regions with fewer restrictions and hence higher diffusivities and MSL. However, while overall MSL showed little change, a decrease in MK was observed. This would suggest that while water molecules were less restricted, there was a concurrent increase in the effective viscosity in the cell environment. Equally likely, the kurtosis could originate from multiple tissue compartments, and its decrease could originate from differential changes in diffusivities in each compartment. The decomposition of kurtosis and non-Gaussian diffusion in the heart remains an open question. Nevertheless, DSI maps show promise as sensitive, if not yet fully understood at the mechanistic level, markers of change in the myocardium.

Cardiac diffusion studies have thus far correlated measured diffusion parameters to various physiological states such as infarct (Mekkaoui et al., 2012, Sosnovik et al., 2009a, Sosnovik et al., 2009b, Strijkers et al., 2009) and hypertrophy (McGill et al., 2012). A major advance would be to extend the characterisation of myocardial tissue, based on diffusion parameters, to clinically relevant biological parameters. Using appropriate models, studies in the brain, for example, have used non-Gaussian diffusion MRI to probe tissue properties such as axon diameter (Barazany et al., 2009), or neurite density (Jespersen et al., 2010) and dispersion (Zhang et al., 2011), which have been implicated in a range of neurodegenerative diseases. Others have used time-dependent diffusion MRI to estimate changes in fibre diameters and permeabilities in skeletal muscle in response to exercise (Kim et al., 2005, Novikov et al., 2012, Sigmund et al., 2014). Undoubtedly, there is strong motivation for estimating cardiac tissue properties such as cell dimensions and shape, density of heterotypic cell types, permeabilities and volume fractions of tissue, micro-vasculature, extracellular space or the conduction system, to name but a few. These all have the potential for improving the characterisation of tissue remodelling, and they could be validated directly using independent non-MRI methods in the experimental setting (Bishop et al., 2010, Bordas et al., 2010, Bueno-Orovio et al., 2014, Hales et al., 2012). These estimates could deepen our understanding on the microstructural changes underlying a host of clinically relevant processes, including ischemia and infraction, load-dependent remodelling, hypertrophy, inflammation, hypoxia and necrosis. Given the complexity of these dynamic changes in tissue architecture, it is likely that the development of the models needed for estimation of such tissue properties, will be driven by acquisition methods reflecting both Gaussian and non-Gaussian diffusion, of which DSI is a prime example. Further research therefore aims at clarifying the nature of non-Gaussian diffusion in the heart.

There are practical constraints that limit broader application of DSI at present. The first is the need to sample q-space in an accurate grid-like fashion, whilst accounting for imaging gradients and cross-terms. To address this, one can adjust the diffusion gradient strengths in a prospective manner based on prior calibration scans (Teh et al., 2014b), obtain data with opposite polarity diffusion gradients (Neeman et al., 1991), or employ gridding methods in post-processing to compensate for off-grid q-samples (Wu and Alexander, 2007). We chose the first method, which entailed an additional one-off calibration scan, but avoided doubling the scan time or generating interpolation errors.

A second constraint is the need to sample hundreds of points in q-space. This leads to long acquisition times and currently confines its application in cardiac imaging to *ex vivo* fixed samples. Popular methods of accelerating MRI scans include k-space undersampling and parallel imaging. One class of acceleration techniques that has attracted much recent interest is compressed sensing, which combines random undersampling of data, with an iterative non-linear algorithm that enforces data consistency and transform sparsity (Lustig et al., 2007). Earlier work focused on undersampling in k-space (Doneva et al., 2010). However, researchers were quick to take advantage of the additional dimensionality in diffusion MRI to undersample in q-space (Menzel et al., 2011) and joint k-q-space (Mani et al., 2014) with reports of acceleration factors between 4 and 8. Compressed sensing was also tailored for DSI through the use of adaptive dictionaries (Bilgic at al., 2012) and the optimisation of radial q-sampling methods and sparsifying transforms (Paquette et al., 2014). The development and validation of new methods in compressed sensing are expected to shorten acquisition times and to enhance the potential for practical application of DSI and diffusion MRI in general.

## 5 Conclusions

An MRI-compatible Langendorff perfusion setup with integrated RF coil was developed and used in a 9.4T horizontal bore scanner. The optimised setup enabled MRI of the same living heart in three different deformation states: slack (representing diastole), volume overload (simulating increased venous return), and contracture (mimicking peak systole). Besides improving SNR, and thereby precision of DTI measures, the optimised setup provided better control over experimental parameters including the mechanical state, temperature, perfusate-tissue water balance, and allowed for physiological monitoring. Differences in non-Gaussian diffusion and tractography between slack and contractured hearts were identified using DSI, which also highlighted the presence of apical voxels that contain multiple fibre tracts. DSI is a versatile method that augments our ability to investigate water diffusion in the intact organ. It provides information on Gaussian and non-Gaussian diffusion for the characterisation of changes to tissue microstructure, including complex tissue morphometry and locally prevailing cell orientation, during cardiac deformation.

Beyond describing differences between diffusion parametric maps, much work remains to further develop and validate biophysical models of diffusion that accurately represent the tissue micro-architecture. DSI, with its model-free approach, is well placed to serve as a benchmark for the development of such models, as well as a reference for validation of novel acquisition and post-processing methods. Further advances in acceleration techniques, including compressed sensing, may broaden the application of DSI in the heart. We anticipate that the next phase of development in imaging of freely beating isolated hearts will facilitate a more comprehensive characterisation of cardiac histo-anatomy throughout the cardiac cycle, and open up opportunities for translating basic science research and development to clinical application.

## Editors’ note

Please see also related communications in this issue by Walton et al. (2014) and Prakosa et al. (2014).

## Glossary

3D: Three-dimensional
ADC: Apparent diffusion coefficient
BHF: British Heart Foundation
CF: Circumferential fibres
DTI: Diffusion tensor imaging
DSI: Diffusion spectrum imaging
FA: Fractional anisotropy
FOV: Field-of-view
GFA: Generalised fractional anisotropy
LHF: Left-handed fibres
LV: Left ventricle
MK: Mean kurtosis
MRI: Magnetic resonance imaging
MSL: Mean squared length
PDF: Probability density function
RHF: Right-handed fibres
RF: Radio-frequency
ROI: Region-of-interest
SNR: Signal-to-noise ratio
TE: Echo time
TR: Repetition time
